# Challenges experienced by GPs when providing palliative care in the UK: a systematic qualitative literature review

**DOI:** 10.3399/BJGPO.2022.0159

**Published:** 2023-05-03

**Authors:** Rachelle Jones, Jeremy Dale, John MacArtney

**Affiliations:** 1 Warwick Medical School, University of Warwick, Coventry, UK; 2 Unit of Academic Primary Care, Warwick Medical School, University of Warwick, Coventry, UK

**Keywords:** primary health care, general practice, palliative care, qualitative research

## Abstract

**Background:**

GPs in the UK will face increased palliative care demands in the coming years. Understanding what makes providing palliative care difficult for GPs is an important step to planning future services, but currently there is an absence of synthesised literature addressing this area.

**Aim:**

To identify the range of issues that affect GPs’ provision of palliative care.

**Design & setting:**

A qualitative systematic review and thematic synthesis of studies exploring GPs’ experiences of providing palliative care in the UK.

**Method:**

Four databases (MEDLINE, Embase, Web of Science, and CINAHL [Cumulated Index to Nursing and Allied Health Literature]) were searched on 1 June 2022 to identify relevant primary qualitative literature published between 2008 and 2022.

**Results:**

Twelve articles were included in the review. The following four themes were identified that affect GPs’ experiences of providing palliative care: lack of resources to support palliative care provision; fragmented multidisciplinary team (MDT) approach; challenging communication with patients and carers; and inadequate training to address the complexities of palliative care. Pressures caused by increasing workloads and a lack of staffing combined with difficulty accessing specialist teams impeded GPs’ provision of palliative care. Further challenges were deficiencies in GP training, and a lack of patient understanding or unwillingness to engage in palliative care discussions.

**Conclusion:**

A multifaceted approach focusing on increased resources, improved training, and a seamless interface between services, including improved access to specialist palliative teams when necessary, is needed to address the difficulties that GPs face in palliative care. Regular in-house MDT discussion of palliative cases and exploration of community resources could generate a supportive environment for GPs.

## How this fits in

GPs face increasing palliative care workloads in the UK owing to an ageing population. Previous literature has identified specific barriers that GPs face in palliative care provision such as symptom management or engaging with specialist services. However, to the authors’ knowledge, there is an absence of synthesised literature exploring the challenges that GPs face when providing palliative care from a holistic perspective. This systematic review finds that a lack of resources, fragmented MDT approach, and training deficiencies all impact on GPs’ ability to provide palliative care. It is important to address these issues to support GPs to provide palliative care, and consideration of these factors may be beneficial during future service planning.

## Introduction

Palliative care is the care and support of patients, and their families, with life-threatening illness to improve quality of life through pain and symptom control; emotional, spiritual or psychological support; and arrangement of social care.^1,2^ Palliative and end-of-life care in the UK is closely aligned to primary care and is provided by a range of services, including GPs and community nurses with input from specialist palliative care teams.^2,3^ With an ageing UK population, the need for GP input is likely to grow.^4,5^

GPs have an important role in identifying those in need of palliative care, providing individualised medical management, liaising with specialist teams and families, and supporting carers before and after death.^6^ GPs are well placed to provide such care owing to their proximity to the community, ability to provide home visits, and relationships formed with patients.^7–9^ However, GPs face many challenges including the following: time pressures;^10,11^ difficulties of MDT working;^7,12^ knowledge or skills deficiencies;^11^ and the variety of patient needs in palliative care.^11^ These challenges are likely to impact GPs' capacity to support patients with terminal conditions.^13^ Such challenges must be considered within the context of specific health systems, and may vary between them.^14^ Hence, this literature review aimed to synthesise evidence about GPs’ experiences of issues affecting provision of palliative care in the UK, with the intention of developing recommendations about how to support GPs’ ability to provide palliative care.

## Method

A systematic qualitative literature review was conducted to identify key themes to be synthesised and reported.^15,16^ Preferred Reporting Items for Systematic Reviews (PRISMA) guidelines were followed.^17^ A qualitative approach was employed as this was the most appropriate method to encapsulate the breadth of GPs’ experiences and allow a nuanced description and analysis of these. The UK focus of this review aimed to minimise the impact of international variability in primary and palliative care provision on GPs’ experiences; for example, private health insurance systems in the US and the Netherlands.^18^

### Search strategy

An electronic search was generated on 1 June 2022 in MEDLINE, Embase, Web of Science, and CINAHL to identify eligible articles published in English between January 2008 and June 2022 (inclusive). The following four main concepts (including synonyms) were used in combination: general practitioners, palliative care, experiences, and qualitative data (complete search strategies are available in Supplementary figures 1–4). A combination of keywords and database-specific subject headings were searched in MEDLINE and Embase; keywords only were searched in Web of Science and CINAHL. The search was refined to UK studies using published search filters.^19,20^

### Data extraction and quality assessment

Title and abstracts of 1232 articles were reviewed by one author (RJ), according to the eligibility criteria (Table 1). If a definite exclusion could not be made, a copy of the full text was reviewed and any queries discussed with a second author (JM). The resulting articles were screened for eligibility at full-text review (Table 1). The following data were extracted from the included studies: study design, sample size, themes identified, and recommendations (Table 2). The quality of included studies was independently assessed by one author (RJ) using the Critical Appraisal Skills Programme (CASP) qualitative research checklist (Supplementary Table S5); any queries were discussed with a second author (JM).

**Table 1.** Eligibility criteria applied during the data extraction process

Inclusion criteria	Exclusion criteria
The study must be published in English language The study must be published between 2008 and 2022 inclusive (the end-of-life strategy was first developed by the Department of Health in 2008)^50^ The study must present primary qualitative data The study must focus on GPs working in the UK (multi-country studies were included if UK participants made up at least 50% of the total participants and the UK data were reported separately) The study must focus on experiences of GPs (articles including other healthcare professionals were included if GPs made up at least 50% of total participants and the experiences of GPs were reported separately)	The publication was a poster, letter, conference abstract, review, or intervention The study focused solely on paediatric palliative care The study was based during the COVID-19 pandemic The study's main focus was also a standalone topic outside of palliative care (for example, advance care planning, as seen in Gott *et al* ^14^).

**Table 2.** Summary of qualitative studies included in the systematic review

Author and title	Design; sample size	Themes	Recommendations
Bowers *et al* 2020.GPs' decisions about prescribing end-of-life anticipatory medications: a qualitative study^30^	Semi-structured interviews;13 GPs	Something GPs can do Getting the timing right Delegating care while retaining responsibility	Improved MDT communication Improved relationships with palliative care nursing staff
Carter *et al* 2017.General practitioners' perceptions of the barriers and solutions to good-quality palliative care in dementia^32^	Postal survey;138 GPs	Lack of knowledge Limited resources Mismanagement of care Poor MDT approach Family support and involvement	Improved education and training Increased funding for staffing Protected time for clinical work Development of an effective MDT Increased respite funding for families
Chen *et al* 2018.GP perceptions of the adequacy of community-based care for patients with advanced heart failure in a UK region (NI): a qualitative study^22^	Semi-structured telephone interviews;24 GPs	Reactive versus proactive approach Access and communication Neglecting conversations Specialist palliative care only a credible option in end stages	Improved community resources Improved communication with specialty services Training of specialist palliative care community nurses in heart failure Clear guidelines to help determine transition to palliative needs
Mitchell *et al* 2013.Defining the palliative care patient: its challenges and implications for service delivery^23^	Semi-structured interviews;8 GPs	Defining the patient with palliative care needs Differences between patients with and without cancer Impact of a palliative care register	A means to 'flag' patients with potential palliative care needs on discharge from hospital
Mitchell *et al* 2016.Providing end-of-life care in general practice: findings of a national GP questionnaire survey^33^	Online questionnaire;516 GPs	Continuity of care Patient and family factors Medical management Expertise and training	Increased time to spend with patients District nurse training in palliative care Improved MDT working Improved communication with out of hours Maintenance of knowledge
Pocock *et al* 2019.Barriers to GPs identifying patients at the end-of-life and discussions about their care: a qualitative study^24^	Interviews;12 GPs	Palliative care registers mostly populated by patients with cancer Prognostication tools not used GPs want help from secondary care Difficult communication with patients	Set of flags for each disease to help identify if a patient is at the end of life More discussion and honesty about death
Selman *et al* 2017.Primary care physicians' educational needs and learning preferences in end-of-life care: a focus group study in the UK^31^	Semi-structured focus groups;10 GPs, 18 GP trainees	Why education is needed Perceived educational needs Learning preferences Evaluation preferences	Mentoring rather than formal training More training in community end-of-life care
Standing *et al* 2017.How can primary care enhance end-of-life care for liver disease? Qualitative study of general practitioners' perceptions and experiences^25^	Semi-structured interviews;25 GPs	The role of the GP Acknowledging and accepting end of life Collaborative care pathways Social relationships and consequences	Improved specialist communication to GPs regarding patient prognosis Better end-of-life care training Appropriate care pathways Psychological support for patients
Taubert and Nelson 2010.'Oh God, not a palliative': out-of-hours general practitioners within the domain of palliative care^26^	Semi-structured interviews;9 GPs	Motivation for out-of-hours work Time-pressure constraints Continuity-of-care impact Isolation within the system	Compulsory written notes in the patients' homes List of contacts for out-of-hours GPs
Taubert and Nelson 2011.Heartsink encounters: a qualitative study of end-of-life care in out-of-hours general practice^27^	Semi-structured interviews;9 GPs	Emotional involvement and 'housekeeping' Heartsink moments	Enhanced end-of-life teaching for out-of-hours GPs
Taubert *et al* 2011. Whatchallenges good palliative care provision out-of-hours? A qualitative interview study of out-of-hours general practitioners^28^	Semi-structured interviews;9 GPs.	Learning and knowledge base Doctor–patient–carer barriers Fear of prescribing and altering doses	N/A
Wyatt *et al* 2021.Delivering end-of-life care for patients with cancer at home: interviews exploring the views and experiences of general practitioners^^29^^	Semi-structured interviews;11 GPs, 7 GP trainees	Difficulty with definitions Importance of communication and managing expectations Complexity in prescribing The unclear role of primary care in palliative care	Need for 'realistic' conversations with families about end of life Improved end-of-life training for out-of-hours GPs Improved MDT working

MDT = multidisciplinary team.

### Data synthesis and analysis

A six-step framework to thematic analysis^21^ was followed, and one author (RJ) used NVivo software (release 1.5.1) to code the text of the 12 included studies line by line. From this coding, themes were generated based on the recurrence of data identified in primary studies, and were modified according to quantity and uniqueness of content, following discussion with a second author (JM). Analysis of the data generated the following four key themes: lack of resources to support palliative care provision; fragmented MDT approach; challenging communication with patients and carers; and inadequate training to address the complexities of palliative care. Recommendations were identified in the literature and noted separately. Views of out-of-hours GPs were reported separately to allow comparison with in-hours service.

## Results

**Figure 1.**
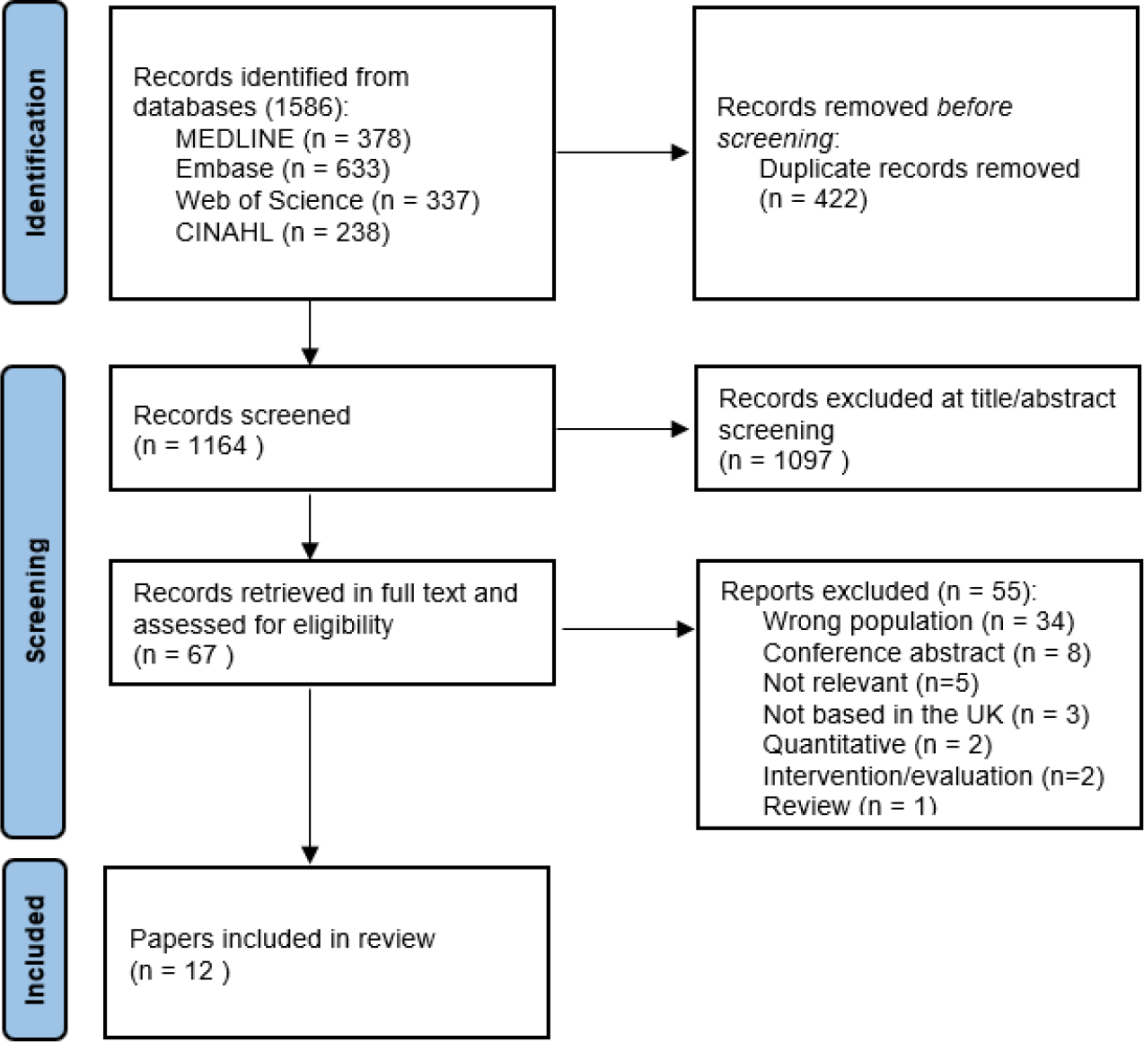
PRISMA flowchart

The literature search identified 1586 citations; 422 duplications were removed, 1097 were excluded during title and/or abstract review, and a further 55 were excluded during full-text review. This meant that 12 articles were included in the analysis (see Figure 1).

### Study characteristics

The design of the 12 studies included the following: interviews (*n* = 9);^22–30^ focus group (*n* = 1);^31^ postal survey (*n* = 1);^32^ and an online questionnaire (*n* = 1).^33^ Five articles focused on in-hours general practice palliative care;^23,24,30,31,33^ three on the out-of-hours context;^26–28^ and the remaining four on specific conditions, which were dementia,^32^ heart failure,^22^ liver disease,^25^ and cancer.^29^ In total, 791 GPs or GP trainees were included, with three articles using the same nine participants.^26–28^ Four articles included participants based in England,^24,25,29,30^ four in Wales,^23,26–28^ two in Northern Ireland,^22,32^ one included participants in England, Scotland and Wales,^33^ and one broadly 'the UK'.^31^

### Themes

#### Lack of resources to support palliative care provision

Five studies found that GPs felt there to be a shortage of staff, particularly district nurses, to care for patients who need palliative care.^22,24,30,32,33^ GPs described a lack of hospice beds,^33^ and geographical variations in access to specialist services^32^ as added challenges. Decision making about when to include patients on palliative care registers (PCR) was affected by these shortages; it was thought to be of little purpose if resources were not available to provide care.^24^ Support from social services in providing home or respite care, and community psychological support, was believed to be insufficient,^22,25,32,33^ at times resulting in patients or families seeking emotional or other support from the GP.^25^

Seven articles described the pressure of GP workloads as a threat to GPs’ time with patients.^22,26,27,29,31–33^ This limited GPs’ ability to address the holistic needs of patients, including conversations concerning resuscitation preferences.^22,29,33^ The short consultation time was felt to be inadequate in the context of palliative care, especially for patients with dementia or multiple comorbidities.^22,31–33^

#### Fragmented MDT approach

Disjointed MDTs and communication issues between services resulted in inconsistent care.^25,30,32,33^ A lack of clarity of the GP's role in palliative care,^29^ difficulty accessing or integration with specialist palliative care teams, and a lack of support from those teams were contributory factors.^22,25,29,32,33^ Poor communication across services was an issue in seven studies, and persistence was needed to overcome barriers hindering quick access to specialists.^22–32,33,32,33^ Inadequate handover from secondary care on patient discharge, specifically regarding prognostication, affected continuity of care.^22,24,25^ This could lead to GPs approaching prognostic conversations apprehensively owing to uncertainty of the patients’ awareness and understanding.^24,25^ Some GPs desired compulsory prognostication from secondary or tertiary care because of this uncertainty.^25^

#### Challenging communication with patients and carers

Although the importance of palliative care discussions was recognised,^31^ eight studies found that GPs faced difficulty in talking to patients about palliative and end-of-life care needs.^22,24,26,29–33^ Reasons included the following: difficulty initiating conversations;^22^ difficulty discussing prognosis or dying;^29,32^ lack of familiarity with patients;^24^ fear of prematurely labelling patients with non-malignant conditions as requiring palliative care; ^22,24,30,33^and reluctance of patients to engage in discussions.^22,30,31^

Some GPs faced further difficulty when patients or families did not understand their diagnosis or disease course.^22,24,26,29,32^ In some cases this led to unrealistic goals,^32^ leaving GPs to manage expectations.^29^ GPs felt that patients with malignant conditions had a better understanding of their prognosis than those with non-malignant conditions.^24^

#### Inadequate training to address the complexities of palliative care

Some GPs felt that they lacked sufficient training in palliative care to meet the complexities of providing care to these patients.^28,31–33^ Many GPs found palliative care complex and challenging.^25–29,31,32^ Defining palliative care or end-of-life care,^23,29^ and the initial identification of a patient as having palliative care needs, especially in non-malignant conditions, were found to be areas of particular difficulty.^22,24,25,29,30,33^

GPs and trainees in one study reported that palliative care training was largely gained in hospital settings.^31^ Once qualified, GPs in several studies described how they struggled to maintain their end-of-life care competencies due to sporadic exposure to patients with these needs, and reliance on specialist services.^29,31,33^ GPs expressed a lack of confidence providing palliative care, which they felt resulted in further reliance on specialist teams,^29^ unnecessary hospital admissions, and poor symptom control.^31^ Specific areas of difficulty were as follows: drug dosing; use of syringe drivers; and complex symptoms.^29,31,33^ Only three studies mentioned prognostication tools such as Gold Standards Framework;^34^ these were either infrequently used or minimally discussed.^23,24,29,31^

### Out-of-hours GPs’ palliative care provision

GPs working for out-of-hours services felt heightened time pressures when called to patients with palliative care needs due to the busy nature of their shifts.^26^ This hindered their ability to emotionally invest in patients.^27^ The unfamiliarity of patients and carers,^27^and the fleeting nature of out-of-hours consultations left some GPs with a profound fear of harming patients.^26,28^ The isolated nature of out-of-hours work was felt to be incompatible with palliative care,^26^ and the electronic systems in many areas were seen as an obstacle to communication between in- and out-of-hours services.^26,30,33^ Some out-of-hours GPs found the minimal palliative care training, and inability to learn on the job because of lack of follow-up, frustrating.^28^

### Recommendations identified within the literature

Recommendations identified within the literature reviewed are included in Table 2. They covered the need to protect clinical time for patients receiving palliative care, and the need to invest in staffing (GPs, district nurses, home support).^22,32,33^ To promote MDT discussion, the use of a palliative care register was seen as an effective tool,^23^ although a clear inclusion criteria was desired,^24^ while a specialist nurse was thought to be aptly placed to coordinate between primary and secondary care.^25^ Improved and regular palliative care updates, with mentoring from palliative care specialists, was also recommended to improve GPs knowledge and confidence.^25,28,31,32^

## Discussion

### Summary

Twelve studies published between 2008 and 2022 were reviewed, which drew on the experiences of 791 GPs or GP trainees in the UK. There were four key themes that challenged GPs’ ability to provide palliative care both in and out of hours. Resource shortages, including staff and the short consultation time, were significant impediments to GPs addressing the holistic needs of patients with palliative care needs. GPs also described how ineffective communication among the MDT contributed to inconsistent care, specifically, a lack of prognostication information from secondary services hindered GPs’ ability to initiate palliative conversations with patients. The fear of disrupting the doctor—patient relationship, and patients’ lack of knowledge regarding their condition or palliative care, compounded the difficulties faced when communicating with patients. GPs also expressed a lack of confidence identifying and managing complex palliative care needs, and described training needs that are currently inadequately addressed.

### Comparison with existing literature

This review found that a lack of a MDT approach resulted in disjointed patient care. This finding is supported by several earlier studies, which highlighted difficulties faced by GPs in MDT communication regarding management of patients needing palliative care, particularly accessing specialist and palliative teams.^8,11,13,35^ This review emphasises that the need remains for improved information sharing between specialists and GPs in the context of palliative care.^8,11,13,35^ It is notable that the lack of MDT approach has also been found to be an impediment to continuity of palliative care from a patient perspective.^8^ This has, at times, forced patients with palliative needs to take the lead in their care and negotiate between services, especially out of hours.^8^

This review found that GPs face difficulty initially identifying a patient as in need of palliative care, especially in the context of non-malignant conditions. There is confusion regarding definitions, and the terms palliative care and end-of-life care are used synonymously, which may result in patients missing out on palliative care.^14,36,37^ Primary care specific tools, for example, Gold Standards Framework and Daffodil Standards,^38^ may be helpful to aid early identification of patients needing palliative care;^39^ however, this review found that they did not feature strongly in GPs’ experiences and may not be appropriate for all types of patients with palliative care needs, such as those with dementia or heart failure.^40^ Further development of such tools may be needed to enhance their applicability to patients with unpredictable disease trajectories.

This review supports established views that palliative and end-of-life discussions between GP and patient are challenging,^41^ with a fear of causing upset via ineffective or inappropriate communication evident. Although literature suggests that many patients value honesty and timely delivery of such discussions,^41,42^ GPs’ and patients’ ambivalence impedes such conversations.^41,43^ Palliative care training, and the use of prognostic tools are proposed to promote initiation of the discussions;^44,45^ however, this review found such tools to be infrequently used, suggesting that further work is needed to enhance their clinical utility as conversation triggers.

The training gaps highlighted in this review have been previously reported.^46^ A 2016 review found that newly qualified doctors felt ill-prepared to manage patients with palliative care needs due to a lack of comprehensive education.^47^ GPs’ knowledge deficiencies in certain aspects of care, such as symptom management, have been previously identified and a negative link to GPs' confidence established.^11,13^ The difficulty accessing specialist teams likely compounds the lack of confidence, particularly out of hours, and therefore not only affects continuity of care, but also quality of care. Although there has been a recent drive to incorporate palliative care into GP training,^6^ there is a lack of research regarding the implementation and effectiveness of this drive. This suggests that a systematic programme of training and education is still needed to equip not only existing GPs, but also medical students with the skills to provide palliative care and to increase their confidence in doing so.^7,29,46,47^ The opportunity for regular discussions of palliative cases among the community MDT may help to develop a supportive environment and improve confidence.

### Strengths and limitations

This review employed a comprehensive and reproducible search strategy. Focusing within the UK and unrestricted by disease topic, it offers important insights into the range of issues affecting the provision of palliative care in UK primary care. The qualitative method enabled the study to focus on GP accounts of their experiences, allowing for a nuanced understanding of the tensions experienced. However, a limitation of the review was that it did not consider experiences of other MDT members, which is a need that must be addressed when planning service improvement.

Articles focusing on specific interventions, such as advanced care planning or the Gold Standards Framework, were excluded. It was noteworthy that such interventions rarely featured in GPs’ overall experiences, but this may also be an artefact of these interventions being outside of the scope of interest of the studies reviewed. Similarly, studies based outside of the UK were excluded, which may limit the applicability of findings to other settings, and also may have excluded insights that could be valuable in generating recommendations for the UK. Although this was done to reduce impact of variables affecting primary and palliative care provision, it is notable that in the UK's devolved nations (Scotland, Wales, Northern Ireland), different contractual models of primary care are employed and as such palliative care delivery likely varies by nation.^48^ Inclusion of studies based during the COVID-19 pandemic would be beneficial for future planning, as this has likely changed the landscape for primary and palliative care going forwards.^49^

### Implications for practice and research

As challenges faced by GPs in the early literature reviewed appear ongoing, a key policy implication is needed to prioritise community palliative care within primary care, and enable greater investment in resources to attend to GPs’ rising palliative care workload. Palliative care education and training needs to be supported throughout a GP's career, and should include more non-malignant diagnoses and on-the-job training within general practice to maximise its relevance. Further research is needed to identify how palliative specialists and GPs can work better together in the community, including how to improve communication, and the role that palliative care registers might have in facilitating this coordinated working.

In conclusion, GPs face many challenges when delivering palliative care to their patients in the UK. There is a need for improved mechanisms of communication across the MDT with easier access to specialist palliative teams. To support GPs to provide palliative care, training is needed throughout a GP’s career, consistent methods must be used to identify patients in need of palliative care, and there should be investment in primary care resources. As these changes require additional resource allocation, a more immediate action can be taken during regular community MDT discussions of palliative cases. Here, initial investment of GPs’ time to explore and strengthen links with locally available palliative resources could generate an ongoing supportive, collaborative working environment to help GPs to manage the rising palliative care workload in the future. It is important to consider these findings during future service planning.

## Data availability statement

All data relevant to this review are included in the article, uploaded as figures and tables or provided as supplementary information.

## Data

All data relevant to this review are included in the article, uploaded as figures and tables or provided as supplementary information.
